# Biological sex, socioeconomic position, and stress physiology among Native Americans: Findings from the Oklahoma Study of Native American Pain Risk III

**DOI:** 10.1016/j.psyneuen.2026.108027

**Published:** 2026-09-30

**Authors:** Jamie L. Rhudy, Travis S. Lowe, Kayla N. Trevino, Parker A. Kell, Taylor V. Brown, Claudia N. Vore, Brandon W. Jones, Aleiyah Fields, William R. Lovallo, Joanna O. Shadlow

**Affiliations:** a TSET Health Promotion Research Center, The University of Oklahoma Health Sciences, 4502 E. 41st Street, Tulsa, OK, 74135, USA; b Department of Health Promotion Sciences, The University of Oklahoma Health Sciences, 4502 E. 41st Street, Tulsa, OK, 74135, USA; c Department of Psychology, The University of Tulsa, 800 S. Tucker Drive, Tulsa, OK, 74104, USA; d Department of Anthropology and Sociology, The University of Tulsa, 800 S. Tucker Drive, Tulsa, OK, 74104, USA; e Department of Anesthesiology, Washington University in St. Louis School of Medicine, 4925 Children’s Place, St. Louis, MO, 63110, USA; f University of Oklahoma Health Sciences and VA Medical Center, 921 NE 13th Street, Oklahoma City, OK, 73104, USA; g Department of Psychology, Oklahoma State University, 700 North Greenwood Avenue, Tulsa, OK, 74106, USA

**Keywords:** Stress reactivity, Stress recovery, Trier Social Stress Test, American Indian, Cortisol, Autonomic

## Abstract

Native Americans (NAs) experience disproportionate exposure to chronic sociocultural stressors that may shape physiological stress systems and contribute to health disparities. Yet, little research has examined acute stress reactivity, stress recovery, and their predictors in NAs. The present study investigated the independent and joint effects of sex and socioeconomic position (SEP) on sympathetic-adrenomedullary (SAM), hypothalamic-pituitary-adrenal (HPA), and negative affective responses to the Trier Social Stress Test (TSST) in 120 healthy NA adults (74 women). SEP was assessed using objective (household income) and subjective (subjective social status) indicators. SAM (skin conductance level, heart rate, blood pressure), HPA (cortisol), and negative affect were measured during baseline, stress, and recovery. Men displayed robust stress responding across all measures, whereas women showed robust SAM and affective responses but blunted cortisol reactivity. Exploratory analyses suggested this difference was not likely explained by hormonal contraceptive use. Lower income was associated with heart rate blunting in both sexes and cortisol blunting among men. By contrast, cortisol blunting was consistent across the income gradient among women. Lower income was associated with poorer cortisol recovery in both sexes. Subjective social status was unrelated to stress outcomes. These findings suggest that sex and SEP jointly shape stress physiology among NAs and support an intersectionality-informed understanding of stress-system functioning. Future research is needed to further understand how sociocultural and structural stressors become biologically embedded in NA populations and contribute to stress-related health disparities, but to also identify sources of resilience.

## 1. Introduction

Native Americans (NAs) experience significant health disparities, including high rates of cardiovascular disease (CVD; Breathett et al., 2020), diabetes (CDC, 2014), obesity (Bullock et al., 2017), substance use disorders (Herron and Venner, 2023), and early mortality (Indian Health Service, 2019). These disparities are driven in part by structural, historical, and contemporary stressors, including discrimination, cultural oppression, and the enduring effects of historical trauma that increase psychosocial stress burden (Evans-Campbell, 2008; Gone et al., 2019). Such stressors can become biologically embedded through repeated activation of physiological stress systems, contributing to stress-related disease risk (Lewis et al., 2021; Thayer et al., 2017; Tiedt and Brown, 2014). Consequently, understanding stress responding in NAs is important because individual differences in stress physiology may represent a pathway linking sociocultural stressors to long-term health outcomes.

Importantly, stressors are likely to affect NAs in intersectional ways. Intersectional psychoneuroendocrinology argues that stress physiology is shaped by intersecting social positions within broader systems of inequality and power (Cook and Mishra, 2026). Thus, understanding how sex and socioeconomic position (SEP) jointly relate to stress physiology may provide insight into mechanisms through which social adversity becomes biologically embedded.

Stress reactivity is coordinated by the sympathetic–adrenomedullary (SAM) and hypothalamic–pituitary–adrenal (HPA) axes. Although these systems typically operate in unison, their coordination can become disrupted, producing heightened, blunted, or uncoupled reactions (Carroll et al., 2017). Such disruptions are clinically significant and linked to chronic stress exposure (Lam et al., 2019), cardiometabolic risk (Phillips et al., 2013), psychiatric symptoms (George et al., 2025), and allostatic load (Beckie, 2012; Thayer et al., 2017; Tiedt and Brown, 2014).

Sex differences in stress physiology are well established, with women often exhibiting blunted HPA responses relative to men (Kudielka et al., 2000; Liu et al., 2017). Moreover, women exposed to adversity often show blunted HPA, but intact SAM responses, indicating HPA-SAM uncoupling (Goldberg et al., 2023; Metz et al., 2020). This raises the possibility that sex differences in stress physiology reflect not only biological influences, but also differences in stress exposure and social position. Consequently, examining stress physiology among NA women and men may provide insight into how gendered experiences become biologically embedded and contribute to cardiometabolic (e.g., CVD, diabetes, metabolic syndrome; Gerdts and Regitz-Zagrosek, 2019) and psychiatric disorders (e.g., anxiety, depression; Stadtler and Neigh, 2023).

However, sex is only one dimension of social position that may shape stress physiology. SEP influences access to resources, exposure to chronic stressors, and health outcomes (Steptoe et al., 2002), and may therefore alter how stress becomes biologically embedded. Lower SEP is associated with blunting of heart rate (HR), blood pressure (BP), and cardiac output (Musante et al., 2000), and a meta-analysis found that higher SEP is associated with greater cardiovascular response to cognitive stressors (Boylan et al., 2018). Importantly, evidence suggests that SEP does not operate uniformly across social groups. For example, one study found that low SEP was associated with stress blunting among Black youth, whereas it was associated with heightened reactivity among White youth (Jackson et al., 1999). Such findings suggest that the physiological significance of SEP depends on social context. There is also evidence that subjective social status (SSS), a person's self-perceived standing within a social hierarchy, may influence stress physiology independent of objective indicators of socioeconomic resources (e.g., Derry et al., 2013; Gruenewald et al., 2006), although findings have been inconsistent (Euteneuer et al., 2026). Together, these findings suggest that both objective and subjective indicators of SEP may shape stress physiology and that their effects may differ across social positions, making them particularly relevant for understanding stress responding within NA populations.

Importantly, the health consequences of stress depend on both stress reactivity and recovery. Whereas stress reactivity reflects activation of SAM and HPA axes, stress recovery reflects post-stress regulatory processes, including parasympathetic reinstatement, vascular relaxation, and HPA negative feedback (Godoy et al., 2018). Sustained sympathetic activation and incomplete cortisol recovery have been linked to cardiovascular risk, inflammation, and psychiatric symptoms and may be as important as reactivity itself in understanding stress-related disease (e.g., Fonkoue et al., 2020; Weber et al., 2010). Previous studies suggest that both sex and SEP may influence recovery, with men showing slower HPA recovery (Liu et al., 2017), women showing slower SAM recovery (Kudielka et al., 2004), and lower SEP being associated with impaired HPA and cardiovascular recovery (Boylan et al., 2018; Hooker et al., 2018). Therefore, assessing both reactivity and recovery provides a more complete characterization of stress-system functioning.

Despite the relevance of sex, SEP, and stress physiology to health disparities, relatively little research has examined these processes among NAs. To our knowledge, only one study has examined both HPA and SAM stress responses in NAs and found evidence of blunted HPA and SAM reactivity to a laboratory stressor relative to non-Hispanic White participants (John-Henderson et al., 2020). However, that study did not examine sex differences, socioeconomic predictors, or stress recovery. Consequently, it remains unclear whether stress physiology varies meaningfully across sex and socioeconomic strata within NA populations.

The present study addresses these gaps by examining sex differences and SEP effects on HPA (cortisol), SAM (skin conductance level [SCL], HR, BP), and subjective (negative affect) stress reactivity and recovery using the Trier Social Stress Test (TSST). Participants were 120 healthy NA adults (74 female) enrolled in the Oklahoma Study of Native American Pain Risk III (OK-SNAP III). SEP was assessed from objective (household income) and subjective (SSS) measures. Based on prior literature, we predicted females would show a blunted stress response relative to males (particularly in HPA responding) and lower SEP would be associated with blunted reactivity and poorer recovery. Consistent with intersectional perspectives, we also examined whether associations between SEP and stress physiology differed by sex. But due to limited prior research, we made no specific predictions regarding sex differences in recovery or differences between objective and subjective indicators of SEP.

## 2. Methods

### 2.1. Participants

The parent study examines risk factors for Native American disparities in chronic pain and health; therefore, only healthy, pain-free participants were enrolled to identify biopsychosocial factors that precede chronic pain onset. This helps rule out treatment effects. To be included, NA participants were required to have self-identified as NA, Indigenous, American Indian, or reported mixed race/ethnicity that included one of these categories. Participants were excluded for: < 18 years of age; current/recent pregnancy; self-reported history of cardiovascular, neuroendocrine, musculoskeletal, or neurological disorders; current chronic pain or headaches; self-reported current substance dependence; medication use that could interfere with testing (e.g., analgesics, anti-depressants, anti-anxiety); current psychotic symptoms (assessed by Psychosis Screening Questionnaire (Bebbington and Nayani, 1995)); serious cognitive impairment (score < 20 on Montreal Cognitive Assessment (Milani et al., 2018; Nasreddine et al., 2005)); peripheral neuropathy (assessed using Michigan Neuropathy Screening Instrument (MNSI) exam score and nerve conduction study (assessed from point-of-care DPNCheck [NC-stat, NEUROMetrix] (Lee et al., 2014)); and inability to read/speak English. Enrollment occurred between February 2023 and October 2025. There were more than 20 different Tribal Nations represented in the study, with the most common being Cherokee, Choctaw, Osage, and Muscogee Creek; however, more than 10% of the sample reported more than one affiliation. Tribal affiliation is not used in analyses at the request of tribal IRBs to respect tribal confidentiality. Table 1 presents participant characteristics by biological sex. Based on the sample size of N = 120 and the slight imbalance in the male:female ratio, this study had 80% power to detect interactions with sex (including Sex × Income) when the effect sizes were ~*f*^2^= 0.07. Thus, the study was adequately powered to detect moderate interaction effects.

### 2.2. Overview of procedures

The parent study included 4 in-person lab visits. Data for the current study were collected during visits 1 and 2. At the 1st visit, written and verbal informed consent were obtained, followed by a thorough assessment of inclusion/exclusion criteria. Resting blood pressure, waist-to-hip ratio, and blood biomarkers were assessed (data not presented). Next, addresses were obtained for geocoding and participants completed a qualitative interview and questionnaire battery (data not presented). The visit concluded with training on how to complete at-home morning and evening saliva collection for diurnal cortisol levels, and how to use a smartphone app that helps verify saliva collection. The second visit assessed stress reactivity using the Trier Social Stress Test (TSST), which is described below. Before the start of the TSST, participants turned in their morning saliva sample. Visit 2 concluded with a questionnaire battery. All procedures were approved by Institutional Review Boards of The University of Tulsa, University of Oklahoma Health Campus, Cherokee Nation, and the Indian Health Service Oklahoma City Area Office. The Cherokee Nation and IHS-OCA IRBs reviewed and approved this manuscript before it was submitted.

### 2.3. Background characteristics

A custom-built demographics and health status questionnaire was used to assess self-reported biological sex, race, ethnicity, age, marital status, education level, household income level, population of current city of residence (to assess rurality), smoking status, and hormonal contraceptive use. BMI was obtained from a medical-grade scale and stadiometer. At visit 1, resting blood pressure and heart rate were assessed three times at 3-min intervals and averaged. Mental health was assessed from the emotional well-being subscale of the Medical Outcomes Study Short Form-36 (SF-36; Ware, Sherbourne, 1992) that measures nervousness, sadness, calmness, and overall emotional well-being over the past 4 weeks. Items are converted to a 0–100 scale and averaged with higher values indicating better mental health (Cronbach’s α current sample =.726). Alcohol use was assessed using the 3-item Alcohol Use Disorders Identification Test-Consumption (AUDIT-C) questionnaire (Bradley et al., 2007). The AUDIT-C assesses frequency, quantity, and heavy episodic drinking to identify hazardous alcohol use and potential alcohol use disorders. Scores range from 0 to 12, with a cutoff of 4 or more for men and 3 or more for women indicating hazardous drinking or possible alcohol use disorder (Bradley et al., 2007). Perceived stress level was assessed using the 10-item Perceived Stress Scale (PSS; Cohen et al., 1983). Scores on the PSS range from 0 to 40, with higher scores indicating greater stress.

### 2.4. Socioeconomic position (SEP)

Household income was used as the primary objective marker of SEP because it represents a household’s immediate ability to meet basic needs (Galobardes et al., 2006). A single item asked participants to report their annual household income (from earnings, unemployment, workers compensation, social security, alimony, child support, etc.) during the preceding calendar year. Ten response options were provided (see Table 1). Exploratory analyses indicated it was reasonably symmetric; thus, it was treated as a continuous variable (Rhemtulla et al., 2012). Education level is often also used as a measure of SEP, so it was examined in exploratory analyses (Supplemental Tables 1 & 2).

Subjective social status was assessed using the MacArthur Scale of Subjective Social Status-USA (SSS-US; Adler et al., 2000). Participants viewed a picture of a ladder representing where people stand in the US in terms of income, education, and occupational prestige. Instructions indicated that individuals at the top of the ladder are “the best off” and those at the bottom are “the worst off.” Participants were asked to mark the rung (1–10) that best reflects their own position. Higher scores represent higher subjective SEP. SSS was only modestly correlated with income (r = .266, p = .004).

### 2.5. Acute stress reactivity: trier social stress test (TSST)

The TSST was used to elicit acute stress. The TSST is a standardized stress induction protocol that reliably activates the HPA and SAM stress arms by exposing participants to uncontrollable, social-evaluative threat. The protocol used was adapted from a previous study (Lovallo et al., 2019). Participants were told their performance would be recorded and compared with other participants to maximize social-evaluative threat.

Sessions always started at 9:00 AM or 1:00 PM to help control for natural fluctuations of stress hormones. Before starting, participants consumed a standardized snack (provided free of charge) to maintain hormone consistency. The meal was followed by a short questionnaire break then a 20-minute resting period during which baseline subjective and physiological data were collected while the participant either watched a neutral video or read a neutral magazine (no cell phones were allowed). The stress phase lasted approximately 45-mins and included both public speaking tasks and mental math. First, participants were prompted to prepare and perform a series of 3 speeches, each with a 4-min preparation period and a 4-minute speaking period. Speech topics included: 1) responding to an erroneous accusation that they were shoplifting, 2) discussing why the participant would be a good candidate for their ideal job, and 3) reasons for the causes of gray hair (outlined in an article they were given). Following the speech tasks, participants performed a series of mental arithmetic tasks that lasted 15-mins. Experimenters maintained neutral affect and provided no performance feedback. A 20-min recovery period followed the stress phase during which participants watched a neutral video or read a neutral magazine. Participants were later debriefed and informed that their responses were not recorded or evaluated.

Blood pressure, skin conductance level (SCL) and electrocardiogram (ECG) were recorded continuously during each phase using Biopac equipment (BIOPAC Systems, Inc.; Goleta, CA). BP was collected using a non-invasive continuous BP device (CNSystems, Austria) that involved placing finger cuffs over the proximal phalanx on the index and middle fingers of the left hand. SCL was recorded from two Ag-AgCl electrodes filled with a.5% saline neutral paste attached to the palmar surface of the middle phalanxes of ring and pinky fingers of the left hand. ECG was collected using two Ag-AgCl electrodes filled with conductive gel attached near the right clavicle and lower left abdomen. All signals were collected using Biopac amplifiers and then digitized using a National Instruments analog-to-digital (A/D) converter (National Instruments; Austin, TX). Signals were processed offline using custom-built LabVIEW software (National Instruments). Prior to averaging, signals were divided into 60-s epochs and inspected. Epochs with poor quality waveforms were rejected (SCL: n = 276, 1.52% of epochs; BP: n = 386, 2.13%; HR: n = 345, 1.90%). The 4-min speech periods were omitted from analysis prior to averaging given that speaking can impact physiology independent of stress. Other epochs were averaged to create 8 periods: 2 baseline, 4 stress, 2 recovery. BP analyses used mean arterial pressure (MAP).

Cortisol was assessed from saliva using the passive drool technique. A total of eight 1 mL samples were collected: 1 upon arrival (not presented), 2 during the baseline phase (10-mins and 20-mins), 3 during the stress phase (16-mins, 24-mins, 39-mins), and 2 during the recovery phase (10-mins and 20-mins). Samples were collected using cryovials (Salimetrics; State College, PA) and then placed in a −20°C freezer until they were shipped to Salimetrics on dry ice for processing. Assays had a sensitivity < 0.007 μg/dL and a range between.012 and 3.000 μg/dL. All samples were analyzed in duplicate and averaged. For analysis, we used these 7 time-points: 2 baseline, 3 stress, and 2 recovery. Only 1 sample was missing (.05% missing).

The 10-item Negative Affect subscale of the Positive and Negative Affect Schedule (PANAS; Watson et al., 1988) was used to assess subjective responses to the TSST. Instructions asked participants to rate how they currently felt on 10 descriptors using a 5-point scale ranging from 0 (“Very Slightly or Not At All”) to 4 (“Extremely”). Items were summed (0–40), with higher scores representing more negative affect. The PANAS was administered at the same 7 time-points that saliva was collected for cortisol. There were no missing negative affect data.

### 2.6. Data analysis

SPSS (IBM; v31.0) was used for all data analyses. Alpha level was set at α< .05 (2-tailed), without a correction for family-wise type I error. Prior to primary analyses, variable distributions were screened using boxplots, histograms, and normality statistics. BMI, AUDIT-C, and Cortisol were Log10 transformed to address positive skew and used in analyses, however, means and SDs for non-log-transformed AUDIT-C and BMI are reported in tables to ease interpretation. Next, outliers were identified using Wilcox’s median absolute deviation procedure (using the recommended 2.24 cutoff) and then winsorized by replacing the outlier value with the next nearest non-outlier value. For analyses examining the associations between sex and SEP with stress reactivity, change scores were created for every dependent variable by subtracting the average baseline score from the average stress period score. The following variables were winsorized: waist-to-hip ratio, and all five stress reactivity change scores. Sex differences in background characteristics were examined with chi-square tests (categorical variables) or independent samples *t*-tests (continuous variables). Cohen’s d is reported as the effect size.

The first set of analyses focus on identifying sex differences in stress responding across time. To do so, multilevel ANOVAs were conducted using the SPSS MIXED procedure and maximum likelihood estimation. These models are statistically powerful and use all available data. Data were kept in long form, with each participant having 8 rows for SCL, HR, and BP, and 7 rows for cortisol and negative affect. These repeated measures were nested within participant, so subject ID was used as the level-2 grouping variable. Repeated measures variance-covariance structure was set to autoregressive order-1 for all variables except for SCL which would not converge so it was set to DIAGONAL. The independent variables were sex (male vs. female) and phase (baseline, stress, recovery), making these 2 × 3 multilevel ANOVAs. In the event of a significant Sex × Phase interaction, simple effects tests were calculated. No effect sizes are provided for these models because there is no agreed upon way to calculate them when there is complex covariance structures. To examine the role of hormone contraceptive use, exploratory analyses were conducted using similar models, but the sex variable had 3 levels (male, female-contraceptive, female-no contraceptive).

The second set of analyses allowed us to examine the joint effect of sex and SEP on stress reactivity. To do so, change scores (stress period–baseline period) for each outcome served as dependent variables in regression models using maximum likelihood estimation (SPSS MIXED). Each model included sex, income, SSS-US, Sex × Income, and Sex × SSS-US as predictors, allowing us to compare the relative effects of income and SSS. Income and SSS-US were z-score transformed prior to forming interaction terms. Sex was coded 0 =male and 1 =female. Pseudo R^2^ values are reported as effect sizes for these analyses.

Simple change scores (e.g., stress – baseline) work well for quantifying stress reactivity, but they are not adequate for calculating recovery because they do not adjust for individual differences in response magnitude (Linden et al., 1997; Miller et al., 2018). For example, the same change between stress and recovery can correspond to different levels of recovery depending on the size of the initial stress response. Instead, percent-based recovery scores are commonly used as a more interpretable, scale-free index (Linden et al., 1997). Recovery was computed as 100 × (stress–recovery) / (stress–baseline), where positive values indicate movement toward baseline. But these scores are unstable when the stress response (stress–baseline) is very small, which creates extreme outliers. To address this, we winsorized them (Wilcox, 2016). These scores were used in the third set of analyses that examined the joint effect of sex and SEP on stress recovery.

## 3. Results

### 3.1. Participant characteristics

Participant characteristics, organized by sex, are presented in Table 1. In general, participants had a mean age of 30.71 years (SD=11.38), mean BMI of 29.01 kg/m^2^ (SD= 7.91), mean waist-to-hip ratio of.87 (SD=.09), mean systolic BP of 120.47 (SD= 13.47), mean diastolic BP of 77.41 (SD= 9.03), mean resting HR of 67.46 (SD= 11.74), mean subjective social status of 4.88 (SD=1.67), mean mental health score of 71.67 (SE=12.15), mean AUDIT-C score of 1.74 (SD= 1.78), and mean PSS score of 24.42 (SD= 7.16). Most participants were single (60%), had at least partial college education (81.3%), lived in an urban cluster (40.8%), and worked full-time (40%). Income was spread somewhat evenly across measured income brackets. There were no significant sex differences in background characteristics, except that men had higher systolic BP, diastolic BP, and waist-to-hip ratios.

### 3.2. Sex differences in stress responsivity over time

Table 2 presents the results of multilevel ANOVA models and Fig. 1 depicts the means and SEMs for the Sex × Phase interactions. There were significant main effects of phase for SCL, BP, HR, and negative affect, indicating that these reactions were increased during the stress period relative to baseline (baseline vs. stress: all ps<.05). Further, SCL and BP were reduced during the recovery phase, relative to the stress phase (stress vs. recovery: ps<.001), but did not return to baseline levels (baseline vs. recovery: ps<.02). By contrast, HR and negative affect returned to baseline values (baseline vs. recovery: ps>.65).

Cortisol did not show a significant main effect of phase, but the Sex × Phase interaction was significant. Simple effects tests for phase indicated cortisol increased during the stress phase for males (baseline vs. stress: p < .001) and did not significantly decrease during the recovery phase (stress vs. recovery: p = .113) but was also not significantly higher than baseline levels (baseline vs. recovery: p = .055). Surprisingly, cortisol significantly decreased during the stress phase for females (baseline vs. stress: p = .042) but the baseline and recovery phases were not significantly different (baseline vs. recovery: p = .687). A significant Sex × Phase interaction was also noted for SCL, but simple effects tests for phase did not identify significant sex differences. Both males and females showed a significant increase in SCL during the stress phase, and SCL was reduced during recovery, but did not completely return to baseline levels (all ps<.001). Further, the simple effects of sex found that males and females did not differ in SCL during baseline, stress, or recovery phases (all ps>.08). Thus, the interaction appears to reflect modest differences in SCL change across phases rather than sex differences at any specific phase. However, these differences were likely too small to detect with follow-up tests.

To rule out that these effects were due to individual differences in females’ use of hormonal contraceptives, the analyses were reran using 3 levels of the independent variable (male, female-contraceptive, female-no contraceptive). These findings are presented in supplemental table 3. The stress-response pattern was similar regardless of hormonal contraceptive use. The only noteworthy finding was that the female-no contraceptive group had overall lower cortisol levels than the other two groups, but their cortisol stress response was blunted like the female-contraceptive group.

### 3.3. The joint effect of sex and SEP on stress reactivity

The results of regression models are presented in Table 3. SSS-US was not significantly related to any outcome, and income was not significantly related to reactivity in SCL, BP, or negative affect. However, income was positively related to HR reactivity, indicating that lower income was associated with HR reactivity blunting for both males and females (Fig. 2A). There were significant main effects of sex and income in the prediction of cortisol reactivity, but they were qualified by a significant Sex × Income interaction. As shown in Fig. 2B, the simple effect of income was significant for males (simple slope=.014, p = .014), but not for females (simple slope=.002, p = .554). Thus, lower income was associated with cortisol reactivity blunting in males, whereas cortisol reactivity was blunted in females across the income gradient. Exploratory analyses with sex, income, SSS, and an Income × SSS interaction as predictors were conducted to examine whether the interplay of the two SEP measures was relevant. The interaction was non-significant in all 5 models (see Supplemental Table 4; ps>.12).

### 3.4. The joint effect of sex and SEP on stress recovery

These results are presented in Table 3. SSS-US was not significantly related to any recovery outcome, and income was not significantly related to SCL, BP, or HR recovery. However, income was positively related to cortisol recovery (Fig. 3), indicating that higher income was associated with a greater proportional movement toward baseline on the cortisol recovery index. Like reactivity analyses, we reran the models with an Income × SSS interaction, but none were significant (see Supplemental Table 5; ps>.088).

### 3.5. Exploratory analyses with education

As shown in supplemental tables 1 & 2, education did not predict any measure of stress reactivity or recovery, nor did it moderate the effect of sex.

## 4. Discussion

To our knowledge, this is the first study to examine SAM, HPA, and affective stress reactivity and recovery during the TSST in NA adults. These findings address an important gap in the stress literature by extending psychophysiological stress models to an understudied population. Guided by an intersectional framework (Cook and Mishra, 2026) we examined the independent and joint influences of sex and SEP on stress reactivity and recovery. Four main findings emerged. First, women exhibited blunted HPA reactivity (cortisol) despite intact SAM (SCL, BP, HR) and affective responses, replicating and extending reports of stress-system dissociation in women (Liu et al., 2017; Metz et al., 2020). Second, income demonstrated a sex-specific association with cortisol reactivity: lower income was associated with cortisol blunting among men, whereas women exhibited blunted cortisol reactivity across the income gradient. Lower income was associated with blunted HR reactivity in both sexes. Third, lower income was associated with poorer cortisol recovery, suggesting socioeconomic disadvantage may influence both stress reactivity and physiological restoration. Fourth, subjective social status (SSS-US) was not associated with stress reactivity or recovery, suggesting conventional measures of perceived social standing may have limited utility for stress physiology among NAs.

### 4.1. Sex differences in stress responding among NAs

The TSST evoked reliable SAM activation (SCL, BP, HR) and negative affect in both sexes, with a partial recovery in SCL and BP and full recovery in HR and negative affect. By contrast, women displayed blunted cortisol reactivity, suggesting a decoupling of HPA and SAM axes among females. This cortisol blunting could stem from higher levels of endogenous opioid activity, which exerts an inhibitory effect on HPA output. Indeed, research has shown that blocking opioid receptors unmasks HPA activation in females (Lovallo et al., 2012). If true, this could have important implications for pain, reward, mood, and motivation in females with cortisol blunting, given the role of endogenous opioids in these processes (Benarroch, 2012). Interestingly, cortisol was not only blunted but showed a significant decrease during the stress period. This likely reflects normal diurnal decline in cortisol rather than stress-induced suppression. Cortisol is expected to decline across both the 9:00 AM and 1:00 PM testing sessions, so the observed decrease may indicate the TSST failed to elicit a cortisol response sufficient to overcome the underlying diurnal decline. Our findings mirror research showing some females have blunted cortisol despite normal autonomic responses. For example, two studies have found that women with PTSD (Metz et al., 2020) or exposure to intimate partner violence (Goldberg et al., 2023) had blunted cortisol responses to the TSST, but showed normal alpha amylase activity (marker of sympathetic activation). This system-specific effect is clinically meaningful because HPA blunting has been linked to allostatic load and stress-related cardiometabolic and psychiatric risk (Carroll et al., 2017; Phillips et al., 2013), even when SAM activation is normal or elevated. Exploratory analyses indicated this profile was independent of hormonal contraceptive use. Moreover, SAM and affective reactivity argue against a failure of the task to evoke stress or a lack of task engagement. It could be due to sex differences in exposure to chronic stressors (Hodes et al., 2024; Stadtler and Neigh, 2023), but there were no sex differences in perceived stress or mental health. However, these measures may not capture gendered, racialized, or culturally specific stressors that could be relevant to HPA functioning. Together, these findings reinforce the need to measure multiple stress-related outcomes, because the absence of cortisol reactivity in women would have been missed had we only collected autonomic and subjective markers.

### 4.2. Intersectional patterning of stress physiology by sex and SEP

Consistent with an intersectional psychoneuroendocrinology framework (Cook and Mishra, 2026), the most notable finding was that income-related differences in cortisol reactivity were evident among men but not women. Lower income was associated with cortisol blunting among men, whereas women exhibited blunted cortisol reactivity across the income gradient. This pattern suggests that socioeconomic resources may relate to HPA-axis functioning differently across social positions among NAs rather than exerting uniform effects.

One interpretation is that income may buffer some stress-related physiological processes among NA men, whereas among NA women, other sources of chronic stress exposure may contribute to attenuated HPA functioning regardless of income. Although the present data cannot identify the mechanisms responsible for this pattern, it is consistent with the possibility that socioeconomic resources do not confer equivalent physiological benefits across gendered social positions. Future work that directly assesses discrimination, caregiving burden, historical trauma, cultural connectedness, and other NA-specific factors is needed to evaluate this possibility.

At the same time, biological mechanisms may also contribute to the sex-specific pattern. The globally blunted cortisol response observed among women mirrors the broader sex differences identified in the present study and is consistent with evidence that females may exhibit attenuated HPA-axis responsivity due to endogenous opioid activity, sex hormones, or sex differences in corticotropin-releasing factor signaling (Hodes et al., 2024; Lovallo et al., 2012). Similar HPA-SAM dissociations have been reported among women exposed to adversity, including women with PTSD and women exposed to intimate partner violence (Goldberg et al., 2023; Metz et al., 2020). Alternatively, the absence of an income association among women may simply reflect a floor effect resulting from the overall lack of HPA responsivity we observed.

By contrast, lower income was associated with blunted HR reactivity in both sexes, suggesting that reduced autonomic responsiveness may represent a more generalized physiological correlate of socioeconomic adversity. Similar findings have been observed outside NA populations, where socioeconomic disadvantage is associated with attenuated cardiovascular responses to laboratory stressors (Boylan et al., 2018; Loeb et al., 2022). Blunted HR reactivity may reflect altered stress-system calibration associated with chronic adversity, although the causal mechanisms underlying this association remain unclear.

SEP differences also emerged in stress recovery. Income was positively associated with cortisol recovery, indicating that higher-income participants exhibited greater proportional movement toward baseline following stress. Conversely, lower-income participants showed less complete cortisol recovery. Because the present study is cross-sectional, these findings should be interpreted as associations rather than evidence that socioeconomic resources directly enhance regulatory capacity. Nevertheless, they suggest that SEP may be related to both the magnitude and resolution of HPA-axis responses. This is significant given that extended cortisol elevations are associated with cardiometabolic risk and inflammation (Beckie, 2012; Thayer et al., 2017; Tiedt and Brown, 2014).

### 4.3. Income vs. subjective social status

Neither subjective social status (SSS-US) nor its interaction with sex was related to reactivity or recovery, and education was similarly unrelated to any outcome. This may mean income better captures material stressors relevant to acute stress dynamics in this population. Although these null findings may reflect a true absence of association, they may also raise questions about whether conventional U.S.-based measures of perceived social standing adequately capture social positions among NAs.

The SSS-US was developed to assess subjective placement within the U.S. social hierarchy (Adler et al., 2000). However, social status among NAs may be influenced by community-specific factors not well represented in conventional status measures, including tribal affiliation, cultural connectedness, kinship networks, and community roles. Thus, income may have been more strongly related to stress physiology because it captures material conditions that directly influence daily stress exposure, whereas social standing may not be adequately represented by the SSS-US measure among NAs. Future studies should evaluate NA-specific conceptualizations of status, social standing, and resource access.

### 4.4. Implications for NA health and disparities

From an intersectional perspective, female-specific cortisol blunting may reflect the biological embedding of gendered stress exposure occurring within historical, structural, and contemporary forms of adversity faced by NA women. Importantly, the absence of an income gradient suggests that physiological vulnerability may not always align with conventional socioeconomic indicators. This finding highlights the potential importance of culturally and socially meaningful factors beyond material resources for understanding both risk and resilience among NA women.

SEP-related findings suggest that socioeconomic disadvantage may shape stress physiology in distinct ways across systems and sexes. Income-linked blunting of HR and cortisol among NA males, together with poorer cortisol recovery among lower-income participants, is consistent with stress-system differences associated with socioeconomic disadvantage. These patterns imply that adverse socioeconomic conditions, such as financial instability, limited access to resources, and chronic environmental demands, become biologically embedded to impair both stress reactivity and regulation.

Together, these results highlight the need for interventions that alleviate socioeconomic strain (e.g., educational opportunities, childcare assistance, food sovereignty, healthcare access) and enhance stress regulation (e.g., mindfulness, community-based resilience programs). They further suggest that policies aimed at improving economic conditions, reducing structural stressors, and strengthening culturally grounded coping resources may reduce stress burden and its downstream health consequences for NAs.

### 4.5. Limitations

Despite the numerous strengths of this study, several limitations must be noted. First, although this is the largest study of NA stress reactivity/recovery to date, power for higher-order interactions was limited (e.g., Sex × Income × SSS). Second, because the primary findings involved sex differences in cortisol responsivity, an important limitation is that menstrual cycle phase, hormonal contraceptive use, menopausal status, and other aspects of hormonal milieu were not measured or incorporated into the analyses. These factors are known to influence HPA-axis responses to psychosocial stress and could have contributed to variability in cortisol reactivity among women. Third, the stressor was laboratory-based and represents only one type of stressor (i.e., social-evaluative). Stress responses can vary by stressor type (social, cognitive, physical), intensity, and cultural relevance, so results may not generalize to other stressors. Fourth, the cross-sectional design prevents determining whether altered stress responses reflect pre-existing vulnerability, consequences of chronic stress exposure, or both. Longitudinal work is needed to clarify temporal ordering and potential developmental pathways. Fifth, we used strict inclusion/exclusion criteria to control many confounds; nonetheless, some factors known to affect stress reactivity/recovery were not explicitly measured or modeled (e.g., diurnal timing, sleep patterns, medication use). Sixth, SEP was operationalized by income and subjective social status. Neither measure fully captures structural determinants relevant for NAs, such as tribal economic systems, cultural roles, land-based resources, or community reciprocity. Seventh, due to the number of statistical tests conducted, some results could reflect Type I errors. And finally, NAs in the study were healthy and primarily represented Southern Plains tribes; therefore, results may not generalize to other NAs or individuals with chronic illnesses/pain. Relatedly, our inclusion criteria necessarily resulted in heterogeneity in our sample, so future studies are needed to examine predictors of stress reactivity unique to NA experiences (e.g., historical loss, cultural connectedness, enculturation). Together, these limitations indicate caution is warranted when interpreting these results until they are replicated.

### 4.6. Future directions

Our findings point to the need for direct assessment of intersectional social stressors, including discrimination, historical trauma, caregiving burden, structural inequities, cultural connectedness, and other sociocultural experiences emphasized by intersectional psychoneuroendocrinology frameworks (Cook and Mishra, 2026), many of which were assessed in the parent study but were beyond the scope of the current report. Such measures may help explain why stress physiology differed across sex and socioeconomic strata within this NA sample. Longitudinal studies are needed to determine whether blunted reactivity reflects a pre-existing vulnerability, an adaptive response to adversity, or a consequence of allostatic load. Further, future work should consider other measures of SEP and chronic stress that capture community-specific forms of resource access. Moreover, examining other stressors could identify whether these HPA/SAM patterns generalize across contexts.

Future studies could also benefit from assessment of inflammatory biomarkers, diurnal cortisol slopes, and endogenous opioid activity, to help characterize the mechanisms of HPA blunting. Given the sex-specific patterns we observed, research should also consider other sex-specific moderators (e.g., menstrual cycle, sex hormones), as well as gendered sociocultural experiences (e.g., caretaking roles for children and elders). Finally, intervention studies are needed to test whether reducing socioeconomic strain and/or enhancing regulatory capacity via culturally-tailored stress-management, economic assistance initiatives, and/or cultural resilience interventions can improve stress reactivity/recovery and improve health trajectories among NAs.

### 4.7. Conclusions

Among NA adults, women showed blunted cortisol reactivity despite intact SAM responses, whereas lower income was associated with blunted cortisol reactivity among men but not women. Lower income was also associated with blunted HR reactivity and poorer cortisol recovery. Together, findings suggest that SEP may relate to stress physiology differently across social positions within NA communities, highlighting the importance of examining both reactivity and recovery and considering intersectional patterning of stress-system functioning.

## Supplementary Material

1

## Figures and Tables

**Fig. 1.**
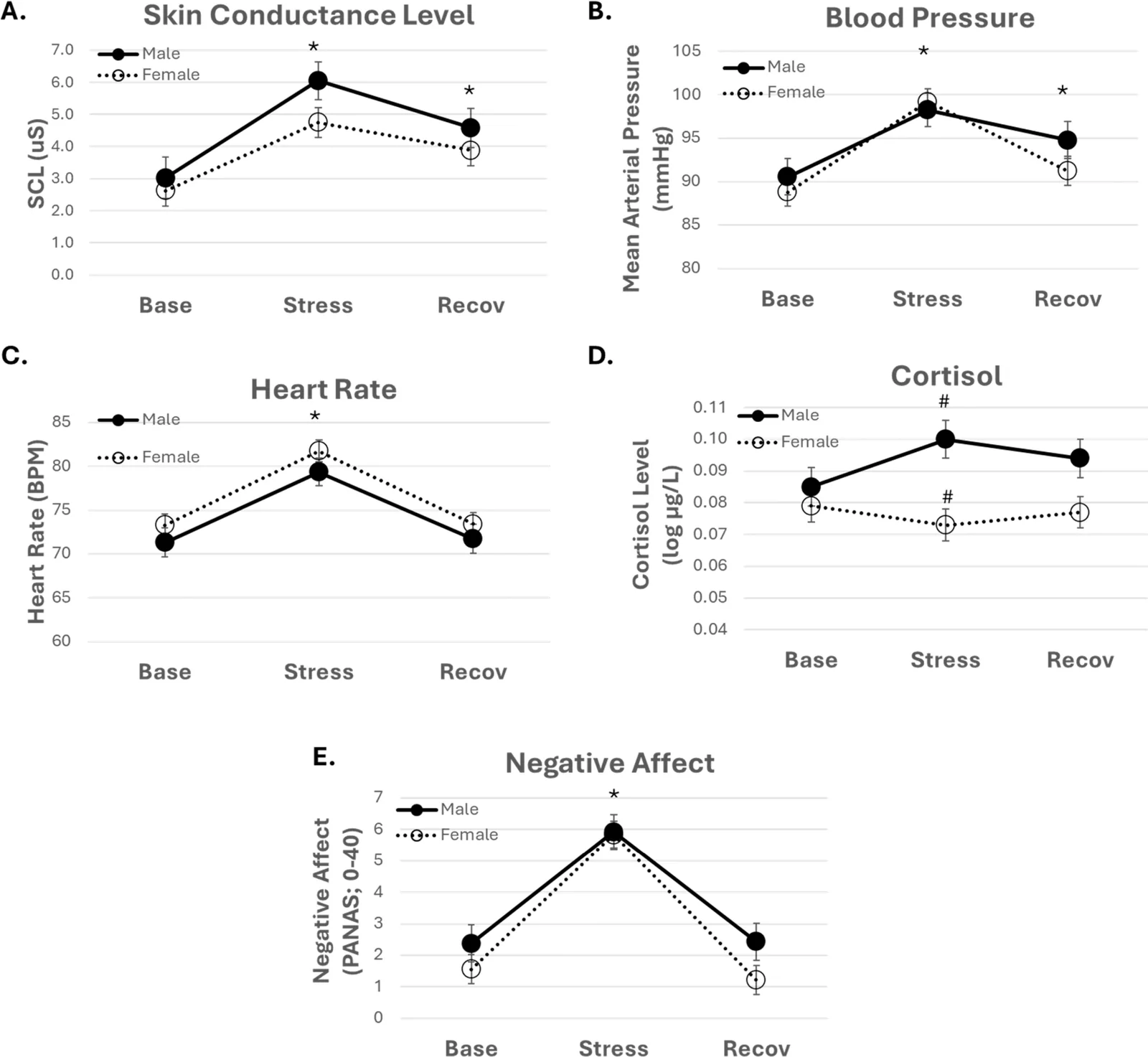
Sex differences in the impact of the Trier Social Stress Test (TSST) on skin conductance (A), blood pressure (B), heart rate (C), salivary cortisol (D), and negative affect (E). Graphs depict the mean (±SEM). *p < .05 for comparison with baseline (averaged across the sexes). ^#^p < .05 for comparison with baseline within each sex (i.e., simple effect of phase).

**Fig. 2.**
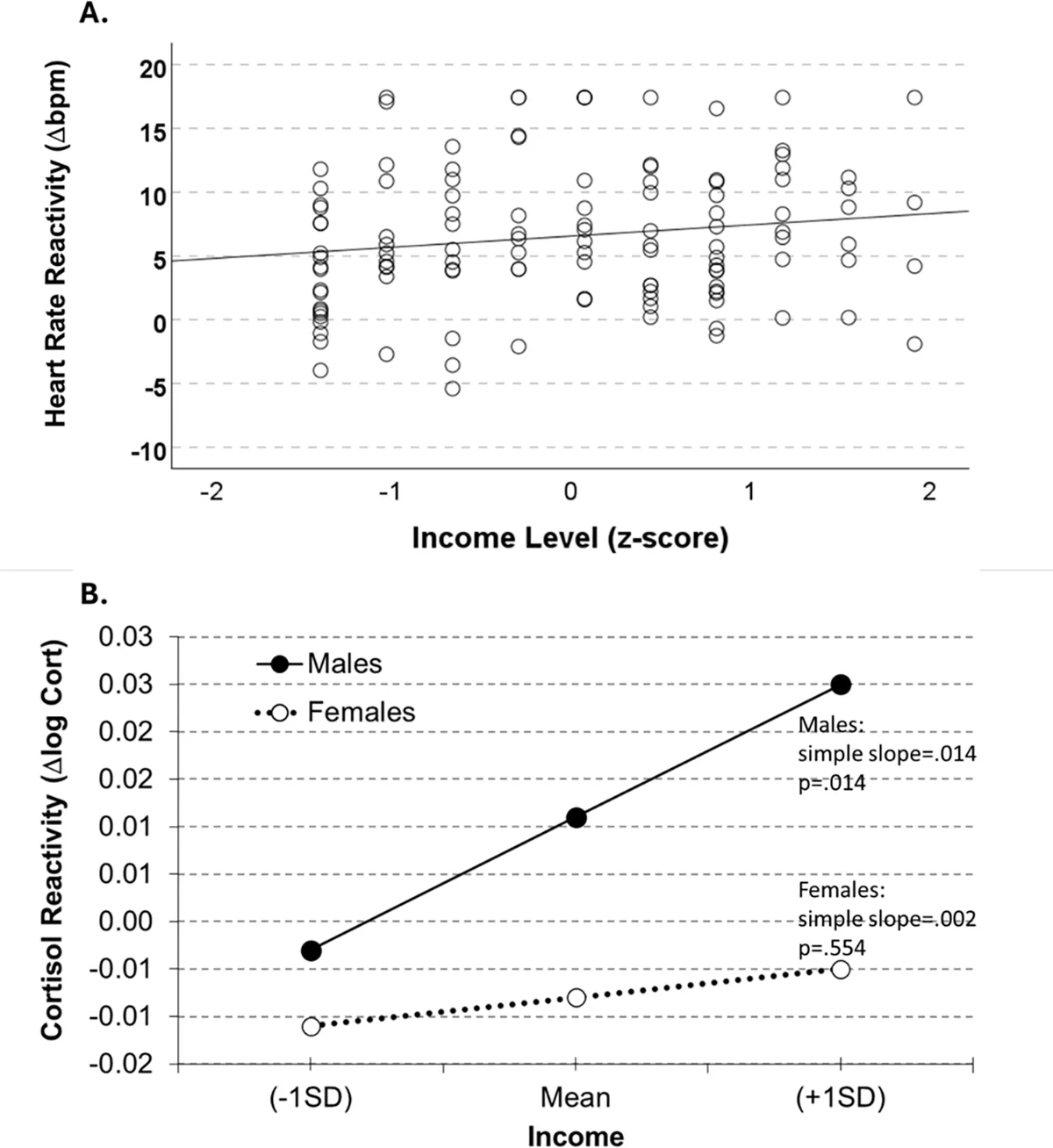
Panel A depicts the relationship between z-transformed income level and heart rate reactivity (stress-baseline). Panel B depicts the interaction between sex and income (z-transformed) in predicting cortisol reactivity (stress-baseline). For males, lower income was associated with blunted cortisol reactivity, whereas cortisol was blunted in females, across the measured income range.

**Fig. 3.**
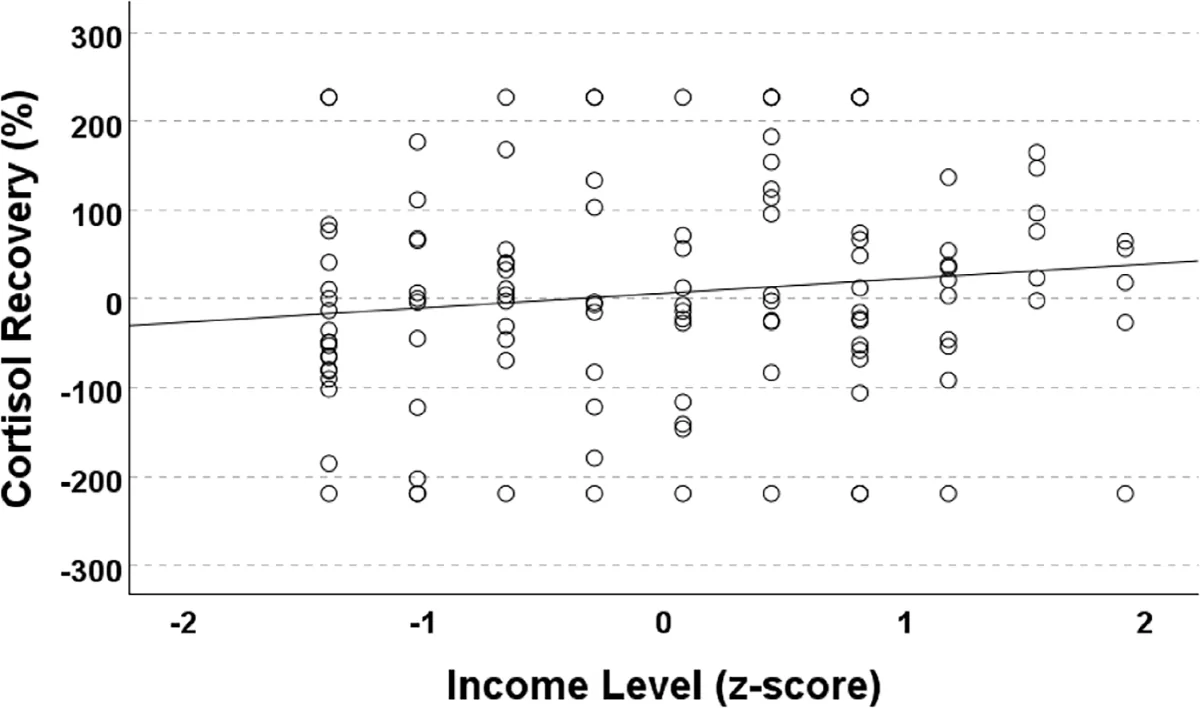
The relationship between z-transformed income level and percent cortisol recovery. Positive percentages are associated with a better recovery. Lower income was associated with poorer cortisol recovery.

**Table 1:** Participant characteristics by sex assigned at birth.

		Male		Female		
Categorical Variables	N	%	N	%	χ^2^	p-value
**Marital Status**					2.924	0.404
single	31	67%	41	55%		
married	12	26%	21	28%		
cohabitating	2	4%	9	12%		
separated/divorced (no payments)	1	2%	3	4%		
**Education**					10.186	.117
Less than seventh grade	1	2%	0	0%		
Junior high school (9th grade)	1	2%	0	0%		
Partial high school (10th or 11th grade)	1	2%	0	0%		
High school graduate	11	24%	8	11%		
Partial college (at least one year)	15	33%	25	34%		
Standard college or university graduation	14	30%	31	42%		
Graduate/professional training	3	7%	10	14%		
**Employment Status**					1.319	0.725
Work 40 or more hours a week	17	37%	31	41.90%		
Work fewer than 40 h a week	14	30%	21	28.40%		
Unemployed	5	11%	11	14.90%		
Student	10	22%	11	14.90%		
**Income**					4.724	0.858
≤ $9,999	9	20%	11	15%		
$10,000 – $14,999	5	11%	7	10%		
$15,000 – $24,999	4	9%	9	12%		
$25,000 – $34,999	3	7%	8	11%		
$35,000 – $49,999	5	11%	7	10%		
$50,000 – $74,999	7	15%	7	10%		
$75,000 – $99,999	6	13%	11	15%		
$100,000 – $149,999	2	4%	8	11%		
$ 150,000 – $199999	2	4%	4	5%		
≥ $200,000	3	7%	2	3%		
**Urban/Rural**					1.847	0.397
Urban (≥50,000 people)	20	44%	22	32%		
Urban cluster (2500–50,000 people)	17	38%	32	46%		
Rural (<2500 people)	8	18%	15	22%		
**Smoking Status**					2.709	0.258
Non-Smoker	26	59%	52	72%		
Prior Smoker	6	14%	9	13%		
Current Smoker	12	27%	11	15%		
**Hormonal Contraceptive Use**						
Yes			24	32%		
No			50	68%		
Continuous Variable	M	SD	M	SD	t	d
Age (years)	29.150	10.828	31.680	11.673	−1.183	−0.222
BMI (kg/m^2^)	29.013	8.695	29.014	7.447	−0.007	−0.001
Waist-to-Hip Ratio	0.919	0.063	0.840	0.082	5.914*	1.044
Resting Systolic BP (mmHg)	128.094	13.017	115.730	11.476	5.448*	1.023
Resting Diastolic BP (mmHg)	80.609	8.290	75.788	9.771	2.780*	0.522
Resting HR (BPM)	67.065	13.480	67.712	10.597	−0.292	−0.055
Subjective Social Status (SSS; 1–10)	5.068	1.784	4.764	1.597	0.952	0.182
Mental Health (SF-36 EW subscale; 0–100)	72.087	12.234	71.405	12.167	0.298	0.056
Alcohol Use (AUDIT-C; 0–12)	2.217	2.200	1.446	1.396	1.383	0.277
Perceived Stress (PSS; 0–40)	23.544	6.982	24.960	7.255	−1.054	−0.198

Note: BMI=body mass index; BP=blood pressure; HR=heart rate; EW=emotional wellbeing; AUDIT= Alcohol Use Disorders Identification Test-Consumption; PSS=Perceived Stress Scale.

* p < .05

**Table 2:** Results of multilevel ANOVA models examining sex differences in stress reactivity.

SCL Results	df num	df denom	F	p-value
Phase	2	415.731	142.848	< .001
Sex	1	125.817	1.106	0.295
Sex x Phase	2	415.731	5.83	0.003
BP Results	df num	df denom	F	p-value
Phase	2	551.664	40.652	< .001
Sex	1	121.75	0.418	0.519
Sex x Phase	2	551.664	2.37	0.094
HR Results	df num	df denom	F	p-value
Phase	2	508.625	222.43	< .001
Sex	1	120.136	1.057	0.306
Sex x Phase	2	508.625	0.283	0.754
Cortisol Results	df num	df denom	F	p-value
Phase	2	589.382	2.025	0.133
Sex	1	120.327	5.761	0.018
Sex x Phase	2	589.382	11.792	< .001
Neg Aff Results	df num	df denom	F	p-value
Phase	2	473.279	127.366	< .001
Sex	1	118.369	1.323	0.252
Sex x Phase	2	473.279	1.89	0.152

**Table 3:** Results of maximum likelihood regression models predicting stress reactivity (left) and recovery (right) from sex and indicators of objective and subjective socioeconomic position.

	Predicting Stress Reactivity				Predicting Stress Recovery			
		
SCL	Estimate	Lower	95% CI Upper	Pseudo R^2^	Estimate	Lower	95% CI Upper	Pseudo R^2^

Sex	−0.307	−0.930	0.315	0.020	−4.138	−20.160	11.883	0.036
Income	0.247	−0.256	0.749		7.472	−5.435	20.378	
SSS-US	0.083	−0.387	0.554		−7.401	−19.487	4.684	
Sex x Income	−0.278	−0.936	0.380		−13.502	−30.404	3.401	
Sex x SSS-US	−0.099	−0.734	0.535		5.217	−11.078	21.512	
BP	Estimate	Lower	Upper	Pseudo R^2^	Estimate	Lower	Upper	Pseudo R^2^
Sex	2.615	−2.259	7.489	0.017	−4.360	−53.433	44.712	0.009
Income	−1.028	−4.987	2.932		9.936	−29.532	49.403	
SSS-US	−0.828	−4.536	2.880		−13.511	−50.306	23.284	
Sex x Income	0.276	−4.830	5.382		−10.146	−61.169	40.878	
Sex x SSS-US	1.130	−3.827	6.088		3.484	−46.047	53.015	
HR	Estimate	Lower	Upper	Pseudo R^2^	Estimate	Lower	Upper	Pseudo R^2^
Sex	0.487	−1.574	2.549	0.042	−3.893	−31.477	23.691	0.018
Income	1.782*	0.108	3.457		3.721	−18.464	25.906	
SSS-US	−0.197	−1.765	1.372		−4.243	−24.926	16.440	
Sex x Income	−1.497	−3.657	0.662		3.662	−25.019	32.343	
Sex x SSS-US	0.059	−2.038	2.156		12.056	−15.786	39.898	
Cortisol	Estimate	Lower	Upper	Pseudo R^2^	Estimate	Lower	Upper	Pseudo R^2^
Sex	−0.019*	−0.030	−0.009	0.164	−42.456	−88.759	3.848	0.064
Income	0.014*	0.005	0.022		40.139*	2.522	77.756	
SSS-US	−0.004	−0.012	0.004		−3.266	−38.491	31.960	
Sex x Income	−0.011*	−0.023	−0.0001		−36.044	−84.551	12.464	
Sex x SSS-US	0.008	−0.003	0.019		−7.815	−54.907	39.276	
Negative Affect	Estimate	Lower	Upper	Pseudo R^2^	Estimate	Lower	Upper	Pseudo R^2^
Sex	0.908	−0.444	2.260	0.064	−9.151	−28.972	10.669	0.023
Income	−0.766	−1.864	0.332		−9.134	−25.340	7.071	
SSS-US	−0.438	−1.467	0.590		6.088	−10.427	22.602	
Sex x Income	0.696	−0.721	2.112		10.461	−10.322	31.244	
Sex x SSS-US	−0.244	−1.619	1.131		−8.871	−29.890	12.148	

Note: SSS-US=MacArthur Subjective Social Status Scale-United States version.

* p < .05

## Data Availability

The data used in this study include information from Native American participants and are subject to tribal sovereignty and federal protections. Because of these requirements, the dataset is not publicly available. Data may be made available upon reasonable request to the corresponding author. Any request to access the data may require review and approval by the Cherokee Nation and the Indian Health Service Oklahoma City Area Office IRBs. Access will only be granted if all applicable tribal, federal, and ethical guidelines are met.

## Appendix A. Supporting information

Supplementary data associated with this article can be found in the online version at doi:10.1016/j.psyneuen.2026.108027.
