# Supplementary material for: Biological sex, socioeconomic position, and stress physiology among Native Americans: Findings from the Oklahoma Study of Native American Pain Risk III
Source: Psychoneuroendocrinology. Author manuscript; Available in PMC 2026 Nov 2. (PMC13726897; doi:10.1016/j.psyneuen.2026.108027)
Supplement: Supplementary file 1 [file NIHMS2214518-supplement-1.docx]

Supplemental Table 1.

Results of maximum likelihood regression models predicting stress reactivity from sex, education, and SSS

|  |  |  | **95% CI** |  |  |
| --- | --- | --- | --- | --- | --- |
| **ΔSCL Results** | **Estimate** | **Lower** | **Upper** | **p-value** | **Pseudo R^2^** |
| Sex | -0.353 | -1.010 | 0.303 | 0.289 | 0.014 |
| Education | 0.108 | -0.323 | 0.539 | 0.621 |  |
| SSS-US | 0.140 | -0.319 | 0.600 | 0.547 |  |
| Sex x Education | -0.037 | -0.681 | 0.607 | 0.910 |  |
| Sex x SSS-US | -0.177 | -0.791 | 0.437 | 0.570 |  |
|  |  |  |  |  |  |
| **ΔBP Results** | **Estimate** | **Lower** | **Upper** | **p-value** | **Pseudo R^2^** |
| Sex | 3.552 | -1.552 | 8.656 | 0.171 | 0.03 |
| Education | -2.430 | -5.793 | 0.933 | 0.155 |  |
| SSS-US | -1.129 | -4.714 | 2.457 | 0.534 |  |
| Sex x Education | 2.122 | -2.894 | 7.137 | 0.404 |  |
| Sex x SSS-US | 1.245 | -3.536 | 6.026 | 0.607 |  |
|  |  |  |  |  |  |
| **ΔHR Results** | **Estimate** | **Lower** | **Upper** | **p-value** | **Pseudo R^2^** |
| Sex | 0.510 | -1.673 | 2.693 | 0.644 | 0.033 |
| Education | -0.563 | -2.001 | 0.876 | 0.440 |  |
| SSS-US | 0.169 | -1.364 | 1.703 | 0.827 |  |
| Sex x Education | 1.951 | -0.194 | 4.096 | 0.074 |  |
| Sex x SSS-US | -0.420 | -2.465 | 1.624 | 0.685 |  |
|  |  |  |  |  |  |
| **ΔCortisol Results** | **Estimate** | **Lower** | **Upper** | **p-value** | **Pseudo R^2^** |
| Sex | -0.020 | -0.032 | -0.008 | 0.001 | 0.107 |
| Education | 0.005 | -0.003 | 0.013 | 0.222 |  |
| SSS-US | -0.001 | -0.009 | 0.007 | 0.817 |  |
| Sex x Education | -0.007 | -0.018 | 0.005 | 0.259 |  |
| Sex x SSS-US | 0.005 | -0.006 | 0.016 | 0.333 |  |
|  |  |  |  |  |  |
| **ΔNeg Aff Results** | **Estimate** | **Lower** | **Upper** | **p-value** | **Pseudo R^2^** |
| Sex | 1.353 | -0.053 | 2.758 | 0.059 | 0.09 |
| Education | -0.662 | -1.589 | 0.264 | 0.159 |  |
| SSS-US | -0.625 | -1.613 | 0.363 | 0.213 |  |
| Sex x Education | -0.269 | -1.650 | 1.113 | 0.701 |  |
| Sex x SSS-US | 0.056 | -1.261 | 1.373 | 0.933 |  |
|  |  |  |  |  |  |

Note: SSS-US=MacArthur Subjective Social Status Scale-United States version

Supplemental Table 2.

Results of maximum likelihood regression models predicting stress recovery from sex, education, and SSS

|  |  |  | **95% CI** |  |  |
| --- | --- | --- | --- | --- | --- |
| **ΔSCL Results** | **Estimate** | **Lower** | **Upper** | **p-value** | **Pseudo R^2^** |
| Sex | -6.100 | -23.041 | 10.840 | 0.477 | 0.033 |
| Education | 7.378 | -3.678 | 18.434 | 0.189 |  |
| SSS-US | -5.549 | -17.337 | 6.239 | 0.353 |  |
| Sex x Education | -11.142 | -27.886 | 5.603 | 0.190 |  |
| Sex x SSS-US | 1.853 | -13.905 | 17.611 | 0.816 |  |
|  |  |  |  |  |  |
| **ΔBP Results** | **Estimate** | **Lower** | **Upper** | **p-value** | **Pseudo R^2^** |
| Sex | -17.513 | -68.864 | 33.837 | 0.501 | 0.038 |
| Education | 31.886 | -1.211 | 64.984 | 0.059 |  |
| SSS-US | -10.333 | -45.620 | 24.954 | 0.563 |  |
| Sex x Education | -29.447 | -80.261 | 21.368 | 0.253 |  |
| Sex x SSS-US | 0.022 | -47.390 | 47.433 | 0.999 |  |
|  |  |  |  |  |  |
| **ΔHR Results** | **Estimate** | **Lower** | **Upper** | **p-value** | **Pseudo R^2^** |
| Sex | -5.353 | -34.705 | 23.999 | 0.719 | 0.015 |
| Education | 5.706 | -13.213 | 24.625 | 0.551 |  |
| SSS-US | -3.256 | -23.426 | 16.914 | 0.750 |  |
| Sex x Education | -5.565 | -34.611 | 23.481 | 0.705 |  |
| Sex x SSS-US | 13.092 | -14.008 | 40.193 | 0.341 |  |
|  |  |  |  |  |  |
| **ΔCortisol Results** | **Estimate** | **Lower** | **Upper** | **p-value** | **Pseudo R^2^** |
| Sex | -52.940 | -102.094 | -3.785 | 0.035 | 0.051 |
| Education | 21.597 | -10.795 | 53.989 | 0.189 |  |
| SSS-US | 6.096 | -28.439 | 40.631 | 0.727 |  |
| Sex x Education | -2.410 | -50.715 | 45.895 | 0.921 |  |
| Sex x SSS-US | -18.697 | -64.742 | 27.349 | 0.423 |  |
|  |  |  |  |  |  |
| **ΔNeg Aff Results** | **Estimate** | **Lower** | **Upper** | **p-value** | **Pseudo R^2^** |
| Sex | -16.963 | -38.105 | 4.178 | 0.115 | 0.039 |
| Education | 12.749 | -2.699 | 28.198 | 0.105 |  |
| SSS-US | 1.679 | -14.039 | 17.397 | 0.833 |  |
| Sex x Education | -7.546 | -28.918 | 13.827 | 0.486 |  |
| Sex x SSS-US | -4.707 | -24.693 | 15.279 | 0.642 |  |

Note: SSS-US=MacArthur Subjective Social Status Scale-United States version

Table 3. Results of multilevel ANOVA models examining stress reactivity as a function of sex and hormonal contraceptive use

| **SCL Results** | **df num** | **df denom** | **F** | **p-value** |
| --- | --- | --- | --- | --- |
| Phase | 2 | 413.574 | 122.54 | <.001 |
| Sex&Hormone | 2 | 125.824 | 1.09 | 0.339 |
| Sex&Hormone x Phase | 4 | 413.849 | 3.496 | 0.008 |
|  |  |  |  |  |
| **BP Results** | **df num** | **df denom** | **F** | **p-value** |
| Phase | 2 | 553.631 | 41.354 | <.001 |
| Sex&Hormone | 2 | 122.473 | 0.713 | 0.492 |
| Sex&Hormone x Phase | 4 | 552.872 | 2.314 | 0.056 |
|  |  |  |  |  |
| **HR Results** | **df num** | **df denom** | **F** | **p-value** |
| Phase | 2 | 511.173 | 217.183 | <.001 |
| Sex&Hormone | 2 | 120.224 | 0.588 | 0.557 |
| Sex&Hormone x Phase | 4 | 510.473 | 0.608 | 0.657 |
|  |  |  |  |  |
| **Cortisol Results** | **df num** | **df denom** | **F** | **p-value** |
| Phase | 2 | 588.205 | 0.114 | 0.892 |
| Sex&Hormone | 2 | 120.196 | 6.4 | 0.002 |
| Sex&Hormone x Phase | 4 | 588.266 | 6.094 | <.001 |
|  |  |  |  |  |
| **Neg Aff Results** | **df num** | **df denom** | **F** | **p-value** |
| Phase | 2 | 472.882 | 123.156 | <.001 |
| Sex&Hormone | 2 | 118.438 | 0.787 | 0.457 |
| Sex&Hormone x Phase | 4 | 472.882 | 1.963 | 0.099 |

Table 4. Results of maximum likelihood regression models predicting stress reactivity from sex, income, SSS, and Income x SSS

|  |  |  | **95% CI** |  |  |
| --- | --- | --- | --- | --- | --- |
| **ΔSCL Results** | **Estimate** | **Lower** | **Upper** | **p-value** | **Pseudo R^2^** |
| Sex | -.312 | -.937 | .312 | .324 | .013 |
| Income | .059 | -.277 | .394 | .729 |  |
| SSS-US | .025 | -.292 | .341 | .877 |  |
| Income x SSS | .075 | -.238 | .388 | .636 |  |
|  |  |  |  |  |  |
| **ΔBP Results** |  |  |  |  |  |
| Sex | 2.660 | -2.172 | 7.492 | .278 | .032 |
| Income | -.362 | -2.918 | 2.195 | .780 |  |
| SSS-US | -.258 | -2.700 | 2.184 | .835 |  |
| Income x SSS | -1.790 | -4.202 | .621 | .144 |  |
|  |  |  |  |  |  |
| **ΔHR Results** |  |  |  |  |  |
| Sex | .498 | -1.580 | 2.576 | .636 | .025 |
| Income | .858 | -.242 | 1.957 | .125 |  |
| SSS-US | -.180 | -1.230 | .870 | .735 |  |
| Income x SSS | .062 | -.975 | 1.099 | .906 |  |
|  |  |  |  |  |  |
| **ΔCortisol Results** |  |  |  |  |  |
| Sex | -.019 | -.030 | -.008 | <.001 | .130 |
| Income | .007 | .001 | .013 | .021 |  |
| SSS-US | 5.079E-5 | -.006 | .006 | .986 |  |
| Income x SSS | .000 | -.006 | .005 | .913 |  |
|  |  |  |  |  |  |
| **ΔNeg Aff Results** |  |  |  |  |  |
| Sex | .893 | -.450 | 2.236 | .191 | .075 |
| Income | -.485 | -1.196 | .225 | .179 |  |
| SSS-US | -.547 | -1.226 | .132 | .113 |  |
| Income x SSS | .519 | -.152 | 1.189 | .128 |  |
|  |  |  |  |  |  |

Table 5. Results of maximum likelihood regression models predicting stress recovery from sex, income, SSS, and Income x SSS

|  |  |  | **95% CI** |  |  |
| --- | --- | --- | --- | --- | --- |
| **ΔSCL Results** | **Estimate** | **Lower** | **Upper** | **p-value** | **Pseudo R^2^** |
| Sex | -4.133 | -20.264 | 11.998 | .613 | .023 |
| Income | -1.374 | -10.017 | 7.270 | .753 |  |
| SSS-US | -4.640 | -12.803 | 3.523 | .262 |  |
| Income x SSS | 3.892 | -4.170 | 11.955 | .341 |  |
|  |  |  |  |  |  |
| **ΔBP Results** |  |  |  |  |  |
| Sex | -4.135 | -53.136 | 44.866 | .868 | .010 |
| Income | 1.968 | -24.029 | 27.965 | .881 |  |
| SSS-US | -11.573 | -36.192 | 13.045 | .354 |  |
| Income x SSS | 6.632 | -18.219 | 31.483 | .598 |  |
|  |  |  |  |  |  |
| **ΔHR Results** |  |  |  |  |  |
| Sex | -3.283 | -30.960 | 24.394 | .815 | .010 |
| Income | 6.227 | -8.457 | 20.911 | .403 |  |
| SSS-US | 2.432 | -11.473 | 16.338 | .730 |  |
| Income x SSS | -.354 | -14.391 | 13.683 | .960 |  |
|  |  |  |  |  |  |
| **ΔCortisol Results** |  |  |  |  |  |
| Sex | -42.341 | -88.573 | 3.891 | .072 | .066 |
| Income | 23.282 | -1.178 | 47.743 | .062 |  |
| SSS-US | -8.885 | -32.251 | 14.481 | .453 |  |
| Income x SSS | -19.976 | -43.050 | 3.097 | .089 |  |
|  |  |  |  |  |  |
| **ΔNeg Aff Results** |  |  |  |  |  |
| Sex | -9.158 | -29.083 | 10.768 | .364 | .012 |
| Income | -3.111 | -13.555 | 7.333 | .556 |  |
| SSS-US | .611 | -9.669 | 10.890 | .906 |  |
| Income x SSS | 1.389 | -8.433 | 11.210 | .780 |  |
|  |  |  |  |  |  |
